# East Meets West: Home and Community Based Care to Enhance Aging in Place

**DOI:** 10.1093/geroni/igab046.1483

**Published:** 2021-12-17

**Authors:** Takashi Amano, Megumi Inoue

## Abstract

Although the magnitude and rate of aging in Japan and the United States differ, the drastic change in population structure has resulted in common challenges in both countries. One challenge is assisting older people in staying in the community. Enhancement of home- and community-based care allows older people to remain in their homes or spaces of their choice without moving into an institution to receive necessary care. This symposium includes four presentations (two from Japan and two from the U.S.) examining various efforts surrounding home- and community-based care designed to strengthen older people’s abilities to stay in the community. The presenters will cover a wide range of strategies that have been implemented in both countries. The first presenter will describe the development and delivery of a project to expand Arizona’s dementia capable system. The second presenter will describe initiatives of a professional association of geriatrics to promote the concept of aging in place. The third presenter will discuss the Home Hazard Removal Program (HARP), a new home hazard removal and fall risk self-management program delivered in the home by occupational therapists. The fourth presenter will discuss Japan’s national policy priority of promoting the use of home health care within the community-based integrated care system. The symposium will conclude with a review of similarities and differences of various efforts, summarize common goals and challenges, and identify best practices.

